# Dynamic factor models: Does the specification matter?

**DOI:** 10.1007/s13209-021-00248-2

**Published:** 2021-11-23

**Authors:** Karen Miranda, Pilar Poncela, Esther Ruiz

**Affiliations:** 1 Department of Statistics, Universidad Carlos III de Madrid, Madrid, Spain; 2 Department of Economic Analysis: Quantitive Economics, Universidad Autónoma de Madrid, Madrid, Spain

**Keywords:** EM algorithm, Kalman filter, Macroeconomic forecasting, Principal components, State-space model, C32, C55

## Abstract

Dynamic factor models (DFMs), which assume the existence of a small number of unobserved underlying factors common to a large number of variables, are very popular among empirical macroeconomists. Factors can be extracted using either nonparametric principal components or parametric Kalman filter and smoothing procedures, with the former being computationally simpler and robust against misspecification and the latter coping in a natural way with missing and mixed-frequency data, time-varying parameters, nonlinearities and non-stationarity, among many other stylized facts often observed in real systems of economic variables. This paper analyses the empirical consequences on factor estimation, in-sample predictions and out-of-sample forecasting of using alternative estimators of the DFM under various sources of potential misspecification. In particular, we consider factor extraction when assuming different number of factors and different factor dynamics. The factors are extracted from a popular data base of US macroeconomic variables, widely analyzed in the literature without consensus about the most appropriate model specification. We show that this lack of consensus is only marginally crucial when it comes to factor extraction, but it matters when the objective is out-of-sample forecasting.

## Introduction

In recent decades, dynamic factor models (DFMs) have been widely used to represent comovements within large systems of macroeconomic and financial variables, in which the cross-sectional dimension is often relatively large compared with the time dimension; see Stock and Watson (2017) for the importance of DFMs in time series econometrics. DFMs generally assume the existence of a small number of unobserved factors capturing the comovements in the system.^1^ Two main types of procedures for factor extraction are popular in the related literature. First, in many applications, factors are extracted using nonparametric procedures based on principal components (PC), which are attractive because they are computational simple and have well-known theoretical properties. In particular, PC is consistent under mild conditions and, as far as the factors are pervasive and the idiosyncratic dependence is weak, it is robust to the underlying dependence of common factors and idiosyncratic components. As a consequence, PC procedures are very popular for factor estimation and several excellent surveys are available in the literature; see, among others, Bai and Ng (2008a) for a technical survey on the econometric theory for PC. However, when the common factors and/or idiosyncratic components are serially dependent, PC procedures do not use this information and, consequently, they are not efficient.

Alternatively, after casting the DFM as a state-space model (SSM), factors can be extracted using Kalman filter and smoothing (KFS) procedures. One important feature of these procedures is that they open the door to Maximum Likelihood (ML) estimation of the model parameters. Furthermore, KFS is also very flexible, allowing to handle in a straightforward way data characteristics often observed in practice as, for example, missing data, mixed frequencies, seasonal dependencies, nonstationarity or regime-switching nonlinearity. Moreover, KFS procedures are also of interest in empirical applications because they allow incorporating restrictions on the factor loadings, as in multi-level DFMs, or on the idiosyncratic components, and to perform counterfactual exercises; see, for example, Banbura et al. (2011) for multi-level models and Luciani (2015) for counter-factual analysis. However, the main drawback of KFS is that it requires full specification of the dependence of the common and idiosyncratic components, opening the door to potential misspecification; see Poncela et al. (2021) for a very recent survey on KFS for factor extraction in DFMs.

There are few papers looking at the effects of the misspecification of the factors on factor extraction and forecasting and all of them focus on factors extracted using PC. Boivin and Ng (2006) conclude that overestimating the number of factors affects the precision with which they are estimated and the forecasting results, while Barigozzi and Cho (2020a) also conclude that overestimating the number of factors could yield non-negligible estimation errors. In an empirical application forecasting GDP growth for Germany and France, Barhoumi et al. (2013) show that not necessarily more factors imply better forecasting. Gonçalves et al. (2017) analyzing the same data set considered in this paper, conclude that the forecasting ability depends on the specific combination of eight PC factors used in factor-augmented predictive regression. Finally, Breitung and Eickmeier (2011a) conclude that, if the cross-sectional dimension is large, the dynamic properties of the factors are not important for factor extraction and forecasting. This paper contributes to the literature by analyzing the empirical consequences on factor estimation and in-sample predictability and out-of-sample forecasting of extracting factors using not only PC but also KFS under various sources of potential misspecification. In particular, we consider factor extraction and forecasting when assuming different number of factors and different factor dynamics. The analysis is carried out extracting factors from the ubiquitous data base of US macroeconomic variables described by McCracken and Ng (2016) and forecasting some key US macroeconomic magnitudes. Factor extraction procedures have been previously being compared using this data set; see, for example, Poncela and Ruiz (2015) and the references therein. However, as far as we know, the empirical properties of KFS extraction under potential sources of misspecification have not been analyzed before when extracting factors from the same data set; see, Aruoba et al. (2009) for the importance of comparing factor extraction procedures in the context of the same data set. In the particular US macroeconomic data set analyzed in this paper, we show that specifications with more factors and more lags are favored in-sample when looking both at log-likelihood ratio tests and at measures of fit of factor-augmented predictive regressions. However, increasing the number of factors and/or their lag structure does not always lead to an increase in the out-of-sample forecast precision with the out-of-sample mean square forecast errors (MSFEs) being generally minimized when forecasts are based on simple models with one factor extracted using KFS and modelled as an AR(1) process. It is important to note that these results might not be applicable beyond the macroeconomic system considered in this paper. Whether they can be generally applicable is an interesting issue that is beyond our objectives. Careful Monte Carlo experiments could be designed to analyze their general applicability.

The rest of the paper is organized as follows. Section 2 briefly describes the representation of DFMs as SSMs and how factor extraction can be performed by PC and KFS. In Sect. 3, the factors are extracted from a system of US macroeconomic variables under the assumption of serially uncorrelated idiosyncratic components. We analyze the differences in terms of point and interval estimation of factors, in sample prediction and out-of-sample forecasting, when factors are extracted using PC and the KFS under different assumptions on the number of factors and their dynamic dependence. Section 4 concludes the paper.

## Dynamic factor models and factor extraction

For completeness, in this section, we briefly describe the DFM as well as factor extraction based on PC and on KFS.

### The dynamic factor model

The following stationary approximate DFM has been extensively analyzed in the related literature^2^

1 $$\begin{aligned} y_{i t}=\lambda _i^{\prime }F_{t}+\varepsilon _{i t}, \end{aligned}$$

where $y_{i t}$ is the observation of the *i*th variable, for $i=1,\ldots ,N$, at time *t*, for $t=1,\ldots ,T$, $\lambda _i=(\lambda _{i1},\ldots ,\lambda _{ir})^{\prime }$ is the $r\times 1$ vector of unknown factor loadings corresponding to $y_{i t}$, $F_{t}=(f_{1t},\ldots ,f_{rt})^{\prime }$ is the $r\times 1$ vector of unobservable stochastic factors at time *t* and $\varepsilon _{i t}$ is the idiosyncratic component of $y_{i t}$. The number of factors, *r*, is assumed to be known and fixed as the cross-sectional and temporal dimensions, *N* and *T*, respectively, grow. If the idiosyncratic components, are assumed to be cross-sectionally uncorrelated, i.e., their covariance matrix, $\Sigma _{\varepsilon }$ is diagonal, the DFM is known as “*exact*” while, if the idiosyncratic noises are weakly cross-correlated, the DFM is called “*approximate*”.

The DFM in equation (1) can be also written in matrix form as follows

2 $$\begin{aligned} Y=F\Lambda ^{\prime }+\varepsilon , \end{aligned}$$

where *Y* is the $T \times N$ matrix of observations, *F* is the $T\times r$ matrix of factors, $\Lambda$ is the $N \times r$ matrix of factor loadings and $\varepsilon$ is the $T\times N$ matrix of idiosyncratic components. Finally, it is also useful to express the DFM in the following vector form

3 $$\begin{aligned} Y_{t}=\Lambda F_{t}+\varepsilon _{t}, \end{aligned}$$

where $Y_t=(y_{1t},\ldots ,y_{\mathrm{Nt}})^{\prime }$ and $\varepsilon _t=(\varepsilon _{1t},\ldots ,\varepsilon _{\mathrm{Nt}})^{\prime }$ are $N\times 1$ vectors.

### Principal components factor extraction

PC, which is among the most popular factor extraction procedures due to its simplicity and low computational burden together with its good theoretical properties, estimates $\Lambda$ and *F* by minimizing the following sum of squares:

4 $$\begin{aligned} V(r)=(\mathrm{NT})^{-1}\sum _{i=1}^{N}\sum _{t=1}^{T}\left( y_{it}-\lambda _{i}^{'} F_{t}\right) ^{2}. \end{aligned}$$

The factors cannot be individually identified and we can only estimate the space spanned by them. In the context of PC factor extraction, it is popular to assume that $\frac{F^{'}F}{T}=I_r$ and $\Lambda ^{'}\Lambda$ is diagonal with distinct elements in the main diagonal arranged in decreasing order; see Bai and Ng (2013) for a discussion on identification restrictions in DFMs. With respect to the identification of the sign, it could be useful to use external information; see, for example, Geweke and Zhou (1996), who propose determining the sign of a single factor by assuming that its weight in a given variable is positive and Stock and Watson (2016) for an application.

Stock and Watson (2002b) show that, if *r* is known, the serial and cross-sectional correlations of the idiosyncratic components are weak and the factors are pervasive, the space spanned by the estimated PC factors is consistent when both *N* and *T* tend simultaneously to infinity. Later, Bai (2003) shows that, if also $\frac{\sqrt{N}}{T}\rightarrow 0$, the factors are asymptotically normal. If the idiosyncratic noises are further assumed to be serially uncorrelated, then the limiting distributions are independent across *t*. The asymptotic approximation of the mean square error (MSE) of the PC factors at time *t*, ${\tilde{f}}^{\mathrm{PC}}_t$, can be estimated as follows

5 $$\begin{aligned} \mathrm{MSE}_t=\left( \frac{{\tilde{\Lambda }}^{\mathrm{PC}^{\prime }}{\tilde{\Lambda }}^{\mathrm{PC}}}{N} \right) ^{-1}\frac{{\tilde{\Gamma }}_t}{N} \left( \frac{{\tilde{\Lambda }}^{\mathrm{PC}^{\prime }}{\tilde{\Lambda }}^{\mathrm{PC}}}{N} \right) ^{-1}, \end{aligned}$$

where ${\tilde{\Lambda }}^{\mathrm{PC}}$ is the PC estimate of the matrix of loadings and, according to Bai and Ng (2006), ${\tilde{\Gamma }}_t$ can be estimated using the following estimator, which is robust to cross-sectional dependence and heteroscedasticity of the idiosyncratic components

6 $$\begin{aligned} {\widetilde{\Gamma }}_{t}=\frac{1}{n}\sum _{i=1}^{n}\sum _{j=1}^{n}{\tilde{\lambda }}^{\mathrm{PC}}_{i}{\tilde{\lambda }}^{\mathrm{PC}^{{\prime }}} _{j} \frac{1}{T} \sum _{t=1}^{T}{\tilde{\varepsilon }}_{it}{\tilde{\varepsilon }}_{jt}, \end{aligned}$$

where $n=\mathrm{min}[\sqrt{N}, \sqrt{T}]$. Note that the robust estimator in (6) is consistent but requires covariance stationarity with $E(\varepsilon _{it} \varepsilon _{jt})=\sigma _{ij}, \forall t$.^3^

Results on the performance of the asymptotic distribution to approximate the finite sample distribution of the estimated PC factors are scarce; see Ouysse (2006), Poncela and Ruiz (2015) and Maldonado and Ruiz (forthcoming), who show that the uncertainty of PC factors is underestimated when computed using asymptotic results.

### Kalman filter and smoothing factor extraction

Alternatively, factor extraction is often based on KFS. Assume that the vector of common factors, $F_t$, evolves over time according to the following stationary VAR(*p*) model

7 $$\begin{aligned} F_t=\Phi _1 F_{t-1} + \Phi _2 F_{t-2} +\cdots + \Phi _p F_{t-p} + u_t, \end{aligned}$$

where $u_t$ is an $r \times 1$ white noise vector with covariance matrix $\Sigma _u$. Although the vector of idiosyncratic components may have temporal dependence, we will consider that it is white noise.

When a particular specification is assumed for factors, as in (7), DFMs are particular cases of the much larger class of SSMs, in which observable variables are expressed in terms of unobserved or latent variables, which in turn evolve according to some lagged dynamics. It is straightforward to write the DFM as a SSM and, assuming that *r* and *p* as well as all DFM parameters are known, KFS can be implemented to extract the factors, regardless of the cross-sectional dimension, *N*; see Poncela et al. (2021) for a survey on factor extraction based on KFS and a description on how to express the DFM as a SSM when the idiosyncratic components are serially correlated. However, even if *r* and *p* were known, in practice, one needs to estimate the model parameters before KFS algorithms can be run. Assuming that $\Sigma _{\varepsilon }$ is diagonal, i.e., the idiosyncratic components are serially uncorrelated, and assuming normality, estimation of the parameters can be carried out by ML with the Kalman filter (KF) used to compute the innovation decomposition form of the Gaussian likelihood, which is given by

8 $$\begin{aligned} \mathrm{log} L(Y;\Psi ) = - \frac{\mathrm{NT}}{2} \mathrm{log} (2 \pi ) - \frac{1}{2} \sum _{t=1}^{T} \mathrm{log} |\Sigma _t| - \frac{1}{2} \sum _{t=1}^{T} \nu _t^{'} \Sigma _t^{-1} \nu _t, \end{aligned}$$

where the innovation vector, $\nu _t=Y_t-E(Y_t|Y_1,\ldots ,Y_{t-1})$, and its covariance matrix, $\Sigma _t$, can be obtained from the KF and $\Psi$ is the vector of parameters to be estimated, namely the loadings in $\Lambda$, the variances in the main diagonal of the covariance matrix of the idiosyncratic noises, $\sigma _{\varepsilon _1}^2,\ldots ,\sigma _{\varepsilon _N}^2$, the autoregressive parameters of the VAR model for the factors in $\Phi _1$,...,$\Phi _p$ and the parameters in the covariance matrix $\Sigma _{u}$. After imposing the necessary identification restrictions, the log-likelihood can be maximized using numerical optimization with, for example, Newton–Raphson algorithms.^4^ Following Harvey (1989), the identifying restrictions on the parameters considered in this paper are $\lambda _{i,j}=0$ for $j>i$ and $i=1,\ldots ,r$ and $\Sigma _{u}=I_r$.^5^ The resulting estimator is denoted as ML-NO. In this very simple DFM, the main hurdle found in the ML-NO estimator appears when *N* is extremely large, because the number of parameters to be estimated, $r^2\times p + N \times (r+1-\frac{r(r-1)}{2})$, increases with *N*.

Alternatively, given that direct optimization of the log-likelihood in (8) can be infeasible when *N* is large, the ML estimator of the DFM parameters can be obtained by the iterative expectation maximization (EM) algorithm. To simplify the description of the EM algorithm, let us assume that $p=1$, i.e., the factors are specified as a VAR(1) model.^6^ First, starting values for the parameters in ${\hat{\Lambda }}^{(0)}, {\hat{\Sigma }}_{\varepsilon }^{(0)}$ and ${\hat{\Phi }}^{(0)}$, are obtained based on factors and loadings estimated by PC. The starting parameters for the loadings are ${\hat{\Lambda }}^{(0)}={\tilde{\Lambda }}^{\mathrm{PC}}$ while the autoregressive parameters are estimated by the following least squares (LS) estimator

9 $$\begin{aligned} {\hat{\Phi }}^{(0)}=\left( \sum _{t=1}^T {\tilde{f}}^{\mathrm{PC}}_{t-1} {\tilde{f}}_{t-1}^{\mathrm{PC}\prime }\right) ^{-1}\sum _{t=1}^T{\tilde{f}}^{\mathrm{PC}}_t{\tilde{f}}^{\mathrm{PC}\prime }_{t-1}, \end{aligned}$$

and the covariance matrix of the idiosyncratic components is estimated by

10 $$\begin{aligned} {\hat{\Sigma }}_{\varepsilon }^{(0)}=\mathrm{diag} \left\{ \frac{1}{T}\sum _{t=1}^T{\tilde{\varepsilon }}_t{\tilde{\varepsilon }}_t^{\prime } \right\} \end{aligned}$$

where $${\tilde{\varepsilon }}_t=Y_t-{\tilde{\Lambda }}^{\mathrm{PC}} {\tilde{f}}_{t}^{\mathrm{PC}}$$.

The expectation step consists in running the KFS algorithm with the parameters of the DFM substituted by the starting values above to obtain $f^{(0)}_{t|T}$, $P^{(0)}_{t|T}$ and $C^{(0)}_t$, where $f^{(0)}_{t|T}$ and $P^{(0)}_{t|T}$ are the smoothed estimate of $F_t$ and its corresponding estimated MSE, given by the Kalman smoother, and $C^{(0)}_t=E\left[ \left( F_{t}-f^{(0)}_{t|T}\right) \left( F_{t-1}-f_{t-1|T}^{(0)}\right) ^{\prime }\right.$ $\left. |Y_1,\ldots ,Y_T\right]$ can also be obtained by the Kalman smoother by augmenting the state vector to include $F_{t-1}$.^7^ In the maximization step, the parameters of the DFM are estimated as follows

11 $$\begin{aligned} {\hat{\Lambda }}^{(1)}= & {} \sum _{t=1}^TY_tf_{t|T}^{(0)\prime } \left( \sum _{t=1}^T f_{t|T}^{(0)}f_{t|T}^{(0)\prime }+P_{t|T}^{(0)}\right) ^{-1}, \end{aligned}$$

12 $$\begin{aligned} {\hat{\Phi }}^{(1)}= & {} \left( \sum _{t=1}^Tf_{t|T}^{(0)}f_{t-1|T}^{(0)\prime }+C_{t}^{(0)} \right) \left( \sum _{t=1}^T f^{(0)}_{t-1|T}f_{t-1|T}^{(0)\prime }+P_{t-1|T}^{(0)}\right) ^{-1}, \end{aligned}$$

while $\Sigma _{(\varepsilon )}$ is estimated as in (10) with the PC residuals substituted by ${\hat{\varepsilon }}^{(1)}_t=Y_t-{\hat{\Lambda }}^{(0)}f_{t|T}^{(0)}$.^8^ Recall that, for identification purposes, the parameters of the DFM need to be restricted and, therefore, using the restrictions described above, $\Sigma _u=I_r$ does not need to be estimated. Furthermore, denoting by $F^{(S)}$ and $P^{(S)}$, the $T \times r$ matrix of smoothed factors and their corresponding MSE matrix in the steady state, respectively, the restrictions in the loadings can be imposed as follows

13 $$\begin{aligned} vec\left( {\hat{\Lambda }}^{(1)*}\right)= & {} vec\left( {\hat{\Lambda }}^{(1)}\right) \nonumber \\&\quad -\left( R vec\left( {\hat{\Lambda }}^{(1)}\right) -c\right) ^{\prime } \left[ R \left( \left( F^{(S)\prime }F^{(S)} + P^{(S)}\right) ^{-1} \otimes I_N \right) R^{\prime }\right] ^{-1}\nonumber \\&\qquad R \left( \left( F^{(S)\prime }F^{(S)} + P^{(S)}\right) ^{-1} \otimes I_N \right) \end{aligned}$$

where *R* is an $\frac{r(r-1)}{2} \times Nr$ matrix of zeros and ones of the coefficients of the parameters in the restrictions and *c* is a $\frac{r(r-1)}{2}$ vector of zeros for the restrictions considered in this case. Consider, for example, that $r=3$, then the matrix of coefficients of the restrictions is given by the following $3 \times 3N$ matrix14The expectation and maximization steps are iterated until convergence and the corresponding estimator is denoted as ML-EM. The parameters of the DFM with serially and cross-sectionally uncorrelated idiosyncratic components can be estimated by ML-EM regardless of *N*; see, among many others, Stock and Watson (1989, 1991) with $N=4$, Quah and Sargent (1993) with $N=60$ and Proietti (2011) with $N=148$.

If the number of factors, *r*, and their autoregressive lag, *p*, are known and under weak cross-correlation of the idiosyncratic components, Doz et al. (2011) show that the smoothed factors extracted using the TS-LS estimates of the parameters are consistent even if $\Sigma _{\varepsilon }$ is wrongly considered as diagonal when it is not, due to the misspecification error vanishing as *N* and *T* diverge to infinity. Later, Doz et al. (2012) extend the result to the ML-EM.^9^ The $\mathrm{min}\left( \sqrt{N}, T \right)$- consistency and asymptotic normality of the latter factors have been proved by Barigozzi and Luciani (2020b) who derive the conditions under which the asymptotic distribution can still be used for inference in case of mis-specification. Note that normality of the DFM is not required for the asymptotic normality of the factors. Barigozzi and Luciani (2020b) compare the loadings, factors and common components estimated using PC and QML estimators and conclude that, in static DFMs, both procedures are rather similar.

### Forecasting with DFM

When the number of predictors is large, it is very popular to obtain out-of-sample forecasts of the variables of interest using factor-augmented predictive regressions (also known as diffusion indexes as proposed by Stock and Watson 2002a). The one-step-ahead forecast of the *i*th variable in the system is given by

15 $$\begin{aligned} {\hat{y}}_{iT+1|T}=\mu + \sum _{j=1}^{q} \delta _{j} y_{iT-j+1} + \sum _{j=1}^{s} B_j^{\prime } F_{T-j+1} \end{aligned}$$

where $B_j=\left( \beta _{1j},\ldots ,\beta _{rj} \right) ^{\prime }$ are parameters. In practice, the parameters of the diffusion index in (15) are estimated by LS after substituting the factors by the corresponding estimates. When the factors are extracted by PC, Stock and Watson (2002a) show that ${\hat{y}}_{iT+1|T}$ is consistent for $y_{iT+1}$. Bai and Ng (2006) show that, if $\frac{\sqrt{T}}{N}\rightarrow 0$, the LS estimator of the parameters is $\sqrt{T}$ consistent and asymptotically normal. Furthermore, they show that the conditional mean predicted by the estimated factors is $\mathrm{min}[\sqrt{T}, \sqrt{N}]$ consistent and asymptotically normal.^10^ Finally, Bai and Ng (2006) also derive the asymptotic distribution of the forecasts of $y_{iT+1}$, which can be used to construct forecast intervals.^11^

As far as we are concerned, there are no results available on the asymptotic properties of the parameter estimator and forecasts when the factors extracted using KFS are used in (15). Our conjecture is that, if the convergence rates of PC factors and ML-EM factors are the same, so should be the rate of convergence of the conditional mean predicted by (15). All in all, the theoretical results that are known point out to the same convergence rates of the previous estimators and, therefore, it remains an empirical question the true behavior of the different possibilities when analyzing the data.

The usefulness of the factors can be evaluated out-of-sample by comparing the MSFE of the forecasts obtained from the factor-augmented regression in (15) with the following univariate autoregression for $y_{it}$ that does not include the factors

16 $$\begin{aligned} {\hat{y}}^{*}_{iT+1|T}=\mu ^{*} + \sum _{j=1}^{q} \delta ^{*}_{j} y_{iT-j+1}. \end{aligned}$$

In order to test the out-of-sample predictive ability of the factors, one can use the ENC-*F* and MSE-*F* tests as proposed by Gonçalves et al. (2017), who show that the presence of estimated PC factors leads to only minor size distortions of predictive ability tests, although it reduces power relative to the case where factors are observed. The ENC-*F* and MSE-*F* tests are given by

17 $$\begin{aligned} \mathrm{ENC}-F=\frac{\sum _{t=T+1}^{T+H}{\hat{u}}_{1t}\left( {\hat{u}}_{1t}-{\hat{u}}_{2t} \right) }{{\hat{\sigma }}_2^2} \end{aligned}$$

and

18 $$\begin{aligned} \mathrm{MSE}-F=\frac{\sum _{t=T+1}^{T+H}\left( {\hat{u}}_{1t}^2-{\hat{u}}_{2t}^2 \right) }{{\hat{\sigma }}_2^2}, \end{aligned}$$

where *H* is the number of one-step-ahead forecasts, ${\hat{u}}_{1t}=y_{it}-{\hat{y}}^{*}_{it|t-1}$, with ${\hat{y}}^{*}_{it|t-1}$ being the one-step-ahead forecasts obtained from the autoregression in (16) and ${\hat{u}}_{2t}=y_{it}-{\hat{y}}_{it|t-1}$, with ${\hat{y}}_{it|t-1}$ given by the factor-augmented regression in (15). Finally, ${\hat{\sigma }}_2^2=\frac{1}{H}\sum _{t=T+1}^{T+H}{\hat{u}}_{2t+1}^2$. The asymptotic critical values in Clark and McCracken (2001) and McCracken (2007) can be used to test whether the predictive ability of both models is the same using ENC-*F* and $\mathrm{MSE}_F$, respectively.^12^

## Empirical extraction of factors

The forecasting performance of KFS procedures for factor extraction is analyzed, both in-sample and out-of-sample, in the context of the ubiquitous database described by McCracken and Ng (2016), which consists of $N=128$ variables observed monthly from January 1983 up to and including December 2020, with a total of 444 observations *per series*.^13^ Previous to their analysis, the data are transformed to stationarity and outliers and missing observations are dealt with as described by McCracken and Ng (2016). Then, all variables in the system are centered and standardized. The sample period is split into an estimation period from January 1983 to December 2016 ($T=396$) and an out-of-sample forecast period, from January 2017 to December 2020 ($H=48$). The focus of prediction are the stationary transformations of industrial production (IP), inflation, employment and real income; see, among others, Quah and Sargent (1993), Stock and Watson (2002b), Bai and Ng (2008b), Alvarez et al. (2016), McCracken and Ng (2016), Granziera and Sekhposyan (2019) and Stauskas and Westerlund (in press) for the interest in forecasting these variables.

### Determining the number of factors and their dependence

**Fig. 1:**
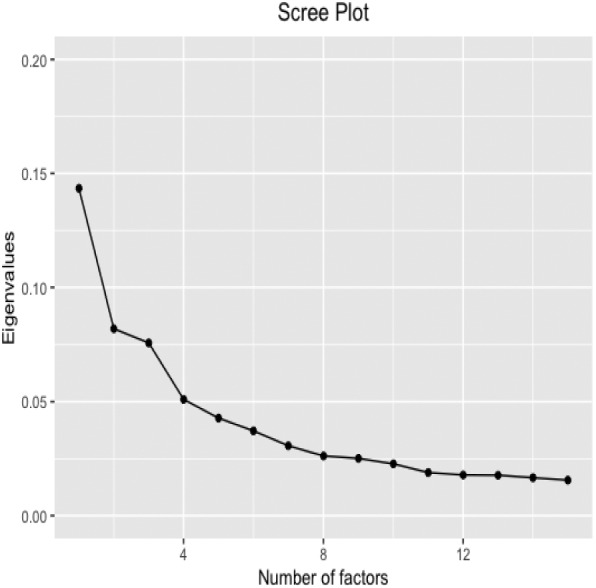
Scree plot of the eigenvalues of the covariance matrix of the US macroeconomic data set

**Table 1:** Determination of the number of underlying factors according to different criteria

	1983–2016
Alessi, Barigozzi, Capasso 1	5
Alessi, Barigozzi, Capasso 2	7
Bai, Ng 1	8
Bai, Ng 2	8
Bai, Ng 3	8
Onatski	1

To determine the number of static factors, we visually inspect the scree plot proposed by Cattell (1966), which appears in Fig. 1; see, for example, Hindrayanto et al. (2016), who also look at the scree plot to determine *r*. The message from the scree plot is not clear with only the presence of one factor being obvious. Alternatively, we also use statistical criteria to determine the number of factors; see Table 1 for a summary of the results. Using the criteria proposed by Alessi et al. (2010), the number of factors is determined to be either $r=5$ or $r=7$. These numbers are in concordance with the related literature analyzing the same data set (observed over different time spans), in which a large number of works determine $r=7$ (Stock and Watson 2005) and Bai and Ng 2007), see also Poncela and Ruiz (2015) and Bennedsen et al. (2021), who chose $r=4$. We also determine *r* using the popular criteria proposed by Bai and Ng (2002), according to which $r=8$; see also Gonçalves et al. (2017), who, in a related application, select 8 factors without using any particular statistical criterion and McCracken and Ng (2016), Demetrescu and Hacioglu Hoke (2019) and Despois and Doz (2020), who also select $r=8$. Moreover, the criteria proposed by Onatski (2010) determines $r=1$; see, for example, Alvarez et al. (2016), who consider the case of $r=1$ factors in this data set. Therefore, there is no agreement about the number of factors that should be used to represent the common movements in the system considered. Stauskas and Westerlund (in press) discuss about the uncertainty relative to the number of factors in this data set. Choi and Jeong (2019) also discuss about the number of factors in this data set and have Monte Carlo results on the difficulty in determining which criterion performs best. Consequently, in order to analyze the effect of the number of factors on the forecasts, we carry out the analysis by assuming three scenarios, namely, $r=1$, $r=3$ and $r=7$.

Once the number of factors, *r*, is determined, factor extraction based on KFS requires assuming a particular lag *p* of the VAR model in (7). It is popular in the related literature, to assume that the dependence of the factors can be represented by VAR(1) models, i.e. by (16) with $p=1$; see, for example, Stock and Watson (2005), Poncela and Ruiz (2015) and Alvarez et al. (2016). However, in practice, the temporal dependence of the factors may be represented by a VAR(*p*) model with $p>1$; see, for instance, Banbura and Modugno (2014), who also consider $p=2$ for a quarterly dataset and Solberger and Spanberg (2020), who also specify $p=2$ for monthly data. In order to chose *p*, we extract a single factor by PC and analyze its correlogram and partial correlogram, which suggest that the factor could be represented by an AR(3) model. Consequently, in order to analyze the effect of *p* on KFS factor extraction and on the corresponding forecasts, we estimate the DFM assuming either that $p=1$ or $p=3$.

### In-sample factor extraction

In this subsection, we first analyze the effect of the choice of *r* and *p* on the properties of factors extracted both by PC and KFS and on the in-sample performance of the factor-augmented predictive regressions in (15).

We first extract one single factor by PC and estimate the corresponding exact DFM with either $p=1$ or $p=3$.^14^ In the latter case, the parameters of the DFM are estimated by TS-LS (PC for the loadings), ML-NO and ML-EM. Table 2 reports a summary of the results. In particular, it reports $\sum _{i=1}^N {\hat{\lambda }}_{i1}^2$, $\sum _{i=1}^N {\hat{\sigma }}_{\varepsilon i}^2$ together with the estimated autoregressive parameters and the MSE of the smoothed factor.^15^ First of all, we can observe that the sums of squared loadings and of idiosyncratic variances and MSE(${\hat{f}}_{t|T}$) are the same regardless of whether the factor is assumed to be AR(1) or AR(3) or whether we estimate the model parameters by ML either using EM or numerically maximizing the log-likelihood. When the Kalman filter is run with the parameters estimated by TS-LS (PC), we can observe that the sum of squared loadings is slightly larger and the sum of idiosyncratic variances is slightly smaller. As a consequence, the Kalman (steady) MSE of the smoothed factor, ${\hat{f}}_{t|T}$, is smaller with an apparent increase in precision as compared with the steady MSE obtained when the parameters are estimated by ML. In any case, it is remarkable that the MSE of the PC extracted factor estimated as proposed in Bai (2003) is 0.01, approximately 5 times smaller than that obtained when the factors are extracted using the KF with ML estimates of the parameters. Furthermore, the implications of the estimation method and specification assumed for the factor are also clear when estimating its dynamic dependence. Consider first the estimated parameters in the model with $p=1$. The ML estimate of the autoregressive parameter (regardless of whether it is estimated maximizing numerically the likelihood, 0.87, or using the EM algorithm, 0.85) is larger than that based on PC, 0.78. Furthermore, note that the slight differences between the ML results obtained when the likelihood is maximized numerically or when using the EM algorithm disappear when $p=3$ is assumed. It seems that when the "true" log-likelihood is maximized its value at the maximum is the same regardless of the procedure used for its maximization. Finally, let us look at the roots implied by the estimated parameters of the AR(3) model. When the parameters are estimated based on the PC factors, the roots are 0.94 and $-0.30\pm 0.35i$ while, if they are estimated by ML, the roots are 0.95 and $-0.27\pm 0.28i$. In both cases, there is a cyclical behavior of the factor with a largest real root when the parameters are estimated by ML. In any case, the persistence of this real root is clearly larger than that obtained when an AR(1) model is assumed for the factor. These differences in the estimated persistence and number of lags of the factor may have implications for forecasting, mainly in periods of changing points because the forecasts adapt quicker if the number of lags is smaller. Finally, Table 2, which also reports the value of the log-likelihood at the maximum for the ML estimates, shows that, although there are not significant differences between the log-likelihood values obtained when the maximization is based on EM or numerical optimization, the difference between the log-likelihood when $p=1$ and $p=3$ is significant, according to the log-likelihood ratio test.

**Table 2:** Parameter estimates (associated with the first factor) of DFMs obtained using TS-LS, ML-NO, and ML-EM when r  =  1, 3 and 7 and p = 1 and 3

	$p=1$			$p=3$		
	TS-LS	ML-NO	ML-EM	TS-LS	ML-NO	ML-EM
***r = *** **1**						
$\sum _{i=1}^N {\hat{\lambda }}_{i1}^2$	18.09	17.72	17.75	18.09	17.75	17.75
$\sum _{i=1}^N {\hat{\sigma }}_{\varepsilon i}^2$	109.87	110.97	110.87	109.86	110.88	110.86
$\hat{\phi _{11}}$	0.78	0.87	0.85	0.34	0.42	0.41
${\hat{\phi }}_{21}$	–	–	–	0.35	0.36	0.36
${\hat{\phi }}_{}31$	–	–	–	0.20	0.14	0.15
MSE (${\hat{F}}_{1t}$)	0.030	0.038	0.044	0.03	0.05	0.05
log-Lik	–	$-69475.1$	$-69485.94$	–	$-69437.7$	$-69438.5$
***r = *** **3**						
$\sum _{i=1}^N {\hat{\lambda }}_{i1}^2$	18.09	–	17.90	18.09	–	17.90
$\sum _{i=1}^N {\hat{\sigma }}_{\varepsilon i}^2$	89.52	–	92.67	89.52	–	92.65
$\hat{\phi _{11}}$	0.78	–	0.86	0.24	–	0.85
${\hat{\phi }}_{21}$	–	–	–	0.49	–	0.31
${\hat{\phi }}_{31}$	–	–	–	0.15	–	$-0.27$
MSE (${\hat{F}}_{1t}$)	0.022	–	0.022	0.024	–	0.027
log-Lik	–	–	–61211	–	–	$-61123.9$
***r =*** **7**						
$\sum _{i=1}^N {\hat{\lambda }}_{i1}^2$	18.09	–	18.03	18.09	–	18.03
$\sum _{i=1}^N {\hat{\sigma }}_{\varepsilon i}^2$	68.76	–	72.73	68.76	–	72.69
${\hat{\phi }}_{11}$	0.78	–	0.74	0.34	–	0.68
${\hat{\phi }}_{21}$	–	–	–	0.45	–	0.29
${\hat{\phi }}_{31}$	–	–	–	0.05	–	$-0.03$
MSE (${\hat{F}}_{1t}$)	0.012	–	0.018	0.012	–	0.028
log-Lik	–	–	$-52948.98$	–	–	$-52744.7$

**Fig. 2:**
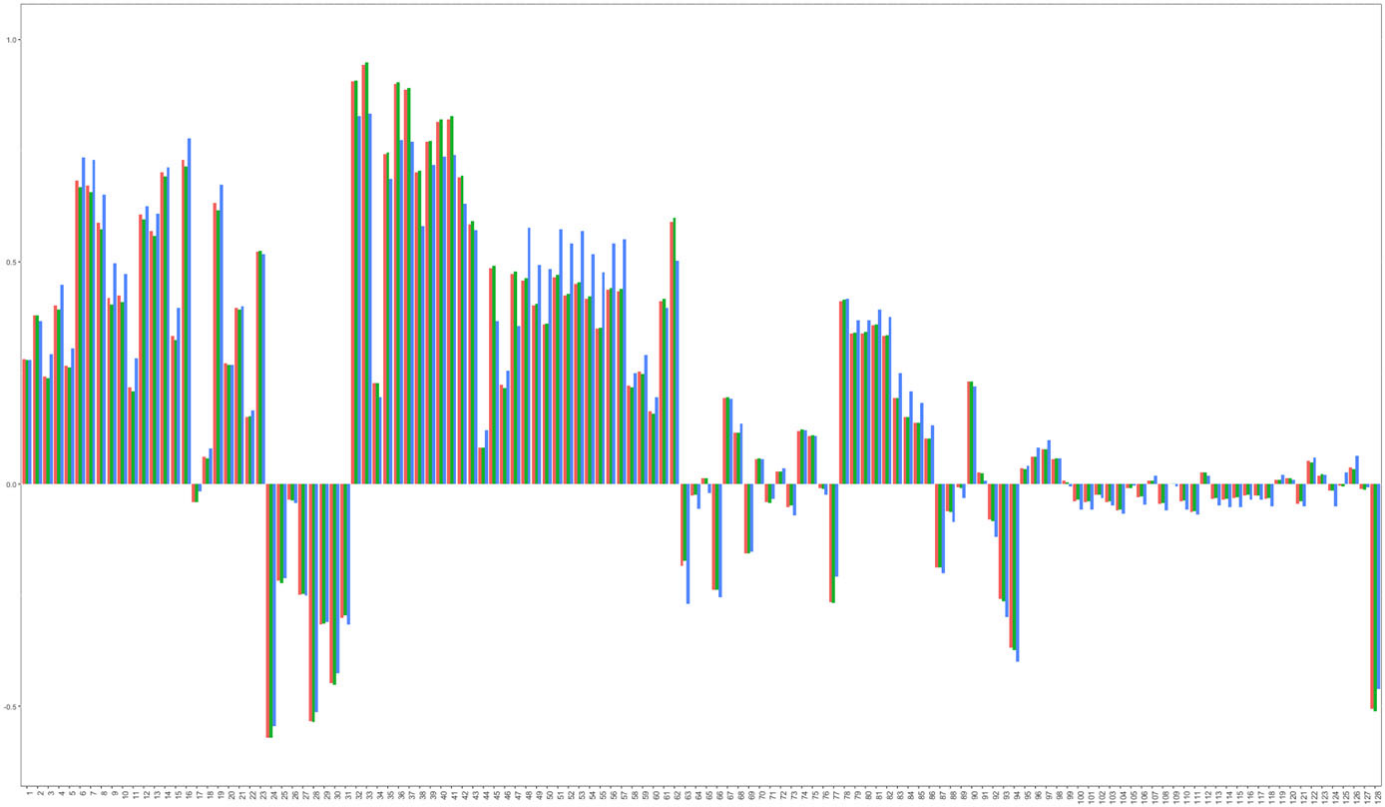
Factor loadings estimated for the set of macroeconomic variables using: (i) PC (blue bars) and (ii) ML with numerical optimization, ML-NO (green bars) and with EM, ML-EM (orange bars) (colour figure online)

**Fig. 3:**
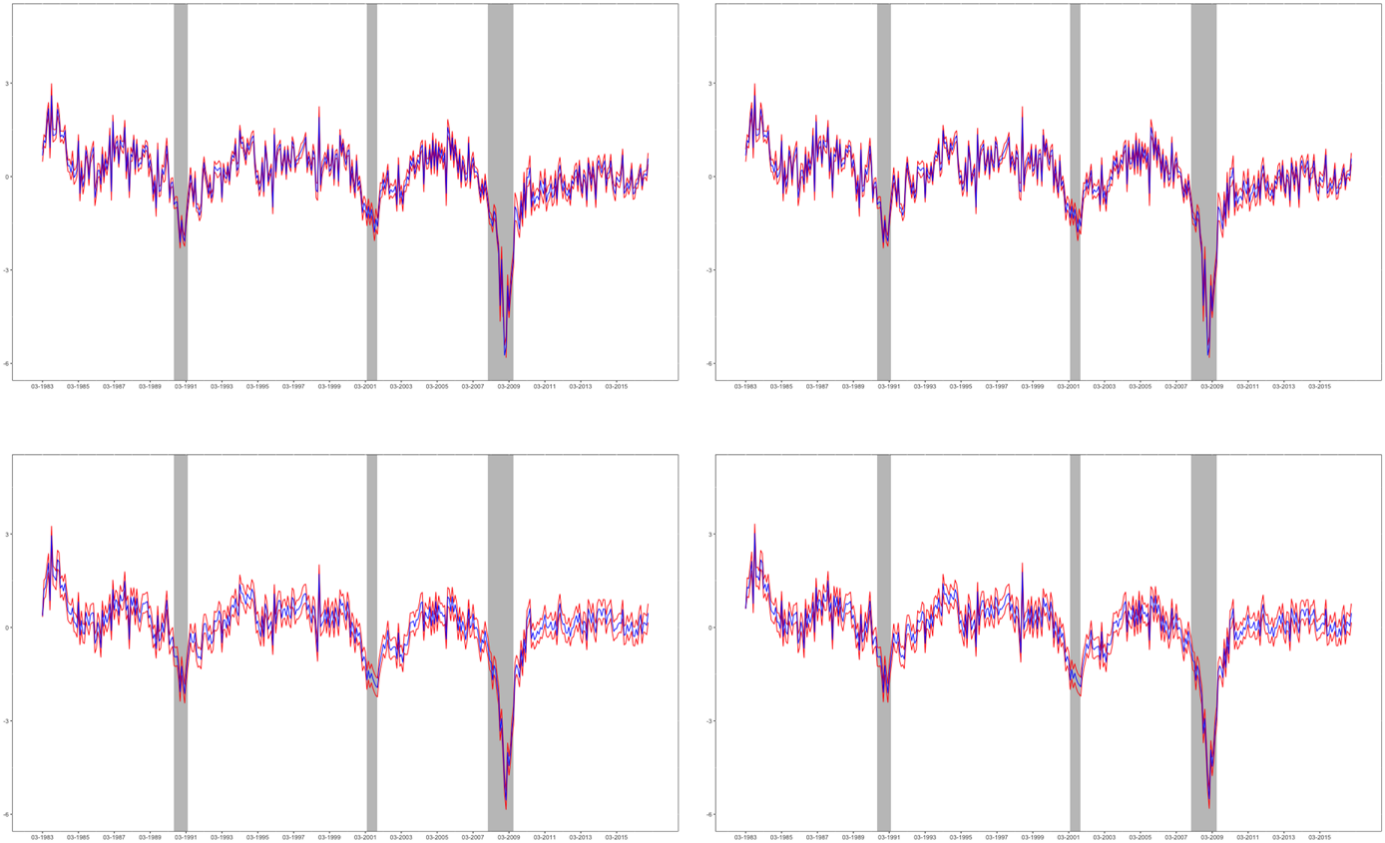
A single factor (blue) extracted from the set of macroeconomic variables using PC (first row) and KFS with EM estimates of the parameters (second row). The first column plots the smoothed factor extracted assuming an AR(1) dependence while in the second column the factor is assumed to be an AR(3) process. The red lines represent the corresponding 95% confidence intervals (colour figure online)

Figure 2, which plots the loadings estimated by PC and by ML, in the latter case, using both numerical optimization and EM, shows that the loadings are similar regardless of the procedure used to estimate them. Furthermore, Fig. 3 plots the factors together with their corresponding 95% confidence intervals obtained by the KFS based on PC and EM parameter estimates reported in Table 2, together with their 95% confidence intervals.^16^ The EM estimated factors have been rotated to be in the same space as those estimated by PC. Figure 3 illustrates that the extracted factors are similar regardless of the particular method implemented to extract them. However, the intervals constructed using ML parameter estimates are clearly larger than those obtained using PC parameters; see also Poncela and Ruiz (2015), who conclude that the asymptotic RMSEs obtained from the asymptotic distribution of the PC factors are unrealistically small.^17^

The conclusions are similar when the factors are extracted assuming either that $r=3$ or $r=7$.^18^ Table 2 shows that the only difference with respect to when *r* was assumed to be 1, is that, obviously, the sum of idiosyncratic variances is now smaller and, consequently, the MSE of the extracted factors is reduced to half.^19^ It is also remarkable that the maximum of the log-likelihood reported in Table 2 is significantly larger when $r=3$ than when $r=1$. Similarly, when we assume that $r=3$ and $p=3$ and the parameters are estimated by TS-LS, we can observe that the estimation results are very similar to those obtained when we assumed that $r=1$ and $p=3$. Looking at the estimated dynamics of the first factor, we can observe that, they are very similar to those estimated when assuming that $r=1$.^20^ In particular, when the parameters are estimated by TS-LS, the roots of the characteristic equation are 0.94 and $0.35\pm 0.20i$, very close to those estimated above. However, the results are somehow different when the parameters are estimated by ML. In this case, the roots are 0.81, $-0.56$ and 0.6, rather different from those obtained when the parameters are estimated by TS-LS and when assuming that $r=1$. Note that the estimation results reported in Table 2 when $r=7$ are similar to those reported when $r=3$.

The sample pairwise correlations between the first factor estimated in the different specifications and estimators considered range from 0.96 to 1.00 when $r=1$ or 3. If $r=7$, some of these correlations fall down to a minimum of 0.8. The minimum correlation, 0.96, is obtained when the factor is extracted assuming that $r=1$ and $p=1$ and estimating the parameters by ML and when it is assumed that $r=3$ and $p=3$ and the parameters of the DFM used to extract the factors are estimated by TS-LS. On the other hand, the maximum correlation, 1.00, is obtained when it is assumed that $r=1$ and $p=3$ and the parameters are estimated by ML either maximizing numerically the likelihood or using the EM algorithm; see also Lewis et al. (2020), who conclude that the factors are robust to whether PC or KFS is implemented for factor extraction when constructing a weekly index of real activity (EWI) based on $N=10$ variables for USA and Breitung and Tenhofen (2011b), who conclude that, in the context of PC factor extraction, the specification of the underlying factors is not important when *N* is large.

Finally, note that the sum of squared loadings (idiosyncratic variances) is larger (smaller) when the parameters are estimated by TS-LS than when estimated by ML and, consequently, the confidence intervals for the factors are larger (and more realistic?) when the parameters are estimated by ML. Assuming a larger number of factors implies reducing the sum of idiosyncratic variances and, consequently, decreasing the MSE of the factors extracted using KF. This result is in concordance with Boivin and Ng (2006) who, in the context of PC factor extraction, conclude that overestimating the number of factors increases the precision of factor estimates (and the forecasts), while underestimating it has the opposite effect (on top of loosing consistency).

### In-sample predictions

**Table 3:** Parameter estimates of factor-augmented predictive regressions for IP based on factors estimated using PC, TS-LS and ML-EM and data observed up to December 2016 together with their corresponding *p*-values in parenthesis, the sample standard deviation of residuals and corrected determination coefficient

	$r=0$	$r=1$					$r=3$					$r=7$				
		PC	$p=1$		$p=3$		PC	$p=1$		$p=3$		PC	$p=1$		$p=3$	
			TS-LS	ML-EM	TS-LS	ML-EM		TS-LS	ML-EM	TS-LS	ML-EM		TS-LS	ML-EM	TS-LS	ML-EM
$\mu$	$-0.007$	$-0.006$	$-0.006$	$-0.006$	$-0.005$	$-0.005$	$-0.006$	$-0.005$	$-0.002$	$-0.005$	$-0.002$	$-0.006$	$-0.004$	$-0.003$	$-0.004$	$-0.003$
	(0.87)	(0.88)	(0.90)	(0.89)	(0.91)	(0.90)	(0.89)	(0.91)	(0.95)	(0.91)	(0.97)	(0.89)	(0.92)	(0.93)	(0.93)	(0.95)
$\delta _1$	0.074	$-0.216$	$-0.256$	$-0.224$	$-0.224$	$-0.191$	$-0.179$	$-0.262$	$-0.128$	$-0.231$	$-0.102$	0.034	0.109	0.015	0.110	0.005
	(0.14)	(0.01)	(0.00)	(0.00)	(0.01)	(0.01)	(0.03)	(0.01)	(0.07)	(0.01)	(0.15)	(0.81)	(0.49)	(0.90)	(0.47)	(0.96)
$\delta _2$	0.164	$-0.094$	$-0.096$	$-0.032$	$-0.113$	$-0.060$	$-0.083$	$-0.125$	$-0.030$	$-0.131$	$-0.046$	$-0.209$	$-0.337$	$-0.184$	$-0.391$	$-0.200$
	(0.00)	(0.23)	(0.23)	(0.64)	(0.16)	(0.39)	(0.32)	(0.17)	(0.66)	(0.15)	(0.50)	(0.14)	(0.04)	(0.13)	(0.01)	(0.10)
$\delta _3$	0.245	0.300	0.306	0.271	0.298	0.262	0.238	0.252	0.254	0.220	0.243	0.287	0.237	0.254	0.190	0.246
	(0.00)	(0.00)	(0.00)	(0.00)	(0.00)	(0.00)	(0.00)	(0.01)	(0.00)	(0.02)	(0.00)	(0.05)	(0.15)	(0.04)	(0.24)	(0.04)
$\delta _4$	0.169	0.408	0.382	0.288	0.381	0.293	0.370	0.427	0.283	0.437	0.287	0.274	0.376	0.394	0.345	0.391
	(0.00)	(0.00)	(0.00)	(0.00)	(0.00)	(0.00)	(0.00)	(0.00)	(0.00)	(0.00)	(0.00)	(0.06)	(0.02)	(0.00)	(0.03)	(0.00)
$\beta _{11}$	–	0.486	0.584	0.671	0.521	0.580	0.322	0.526	0.783	0.421	0.817	0.143	0.212	0.367	0.186	0.381
	–	(0.00)	(0.00)	(0.00)	(0.00)	(0.00)	(0.03)	(0.00)	(0.00)	(0.01)	(0.00)	(0.42)	(0.33)	(0.07)	(0.38)	(0.07)
$\beta _{12}$	–	0.486	0.472	0.359	0.519	0.471	0.467	0.572	0.607	0.705	0.643	0.316	0.584	0.630	0.657	0.725
	–	(0.00)	(0.00)	(0.02)	(0.00)	(0.00)	(0.00)	(0.00)	(0.01)	(0.00)	(0.01)	(0.11)	(0.03)	(0.01)	(0.01)	(0.00)
$\beta _{13}$	–	$-0.220$	$-0.282$	$-0.321$	$-0.267$	$-0.305$	$-0.177$	$-0.433$	$-1.003$	$-0.480$	$-1.097$	$-0.333$	$-0.629$	$-0.747$	$-0.642$	$-0.833$
	–	(0.10)	(0.05)	(0.03)	(0.05)	(0.03)	(0.24)	(0.02)	(0.00)	(0.01)	(0.00)	(0.10)	(0.02)	(0.00)	(0.02)	(0.00)
$\beta _{14}$	–	$-0.603$	$-0.586$	$-0.508$	$-0.588$	$-0.544$	$-0.429$	$-0.441$	$-0.270$	$-0.419$	$-0.239$	0.035	$-0.006$	$-0.176$	0.067	$-0.153$
	–	(0.00)	(0.00)	(0.00)	(0.00)	(0.00)	(0.04)	(0.01)	(0.17)	(0.01)	(0.23)	(0.85)	(0.98)	(0.40)	(0.76)	(0.48)
$\sigma _u$	0.895	0.852	0.852	0.852	0.855	0.856	0.848	0.847	0.841	0.846	0.835	0.817	0.809	0.811	0.795	0.806
${\bar{R}}^2$	0.20	0.27	0.27	0.27	0.27	0.30	0.28	0.28	0.29	0.28	0.30	0.33	0.34	0.34	0.37	0.35

To analyze whether the small differences in the estimated factors have implications on in-sample prediction, we estimate the factor-augmented predictive regressions in equation (15) with $q=s=4$ for each of the four variables to be forecast, namely, IP, inflation, employment and real income, using the factors extracted by the alternative methods considered assuming that $r=1$, 3 and 7 and $p=1$ and 3. Note that, in this application, both *T* and *N* are rather large with $\frac{\sqrt{T}}{N}=\frac{\sqrt{396}}{128}=0.155$ being close to zero and, consequently, using the results in Bai and Ng (2006), we can conclude that the factor estimation uncertainty should be negligible when conducting inference in the factor-augmented regression. Table 3 reports the estimates of the parameters of these regressions for IP growth, $y_t$, together with their corresponding *p*-values obtained under the assumption of homoscedastic forecast errors, $u_{2t}=y_{t}-{\hat{y}}_{t|t-1}$, the sample standard deviation of the corresponding residuals, $\sigma _u$, and the adjusted determination coefficient, ${\bar{R}}^2$. In the case of more than one factor, Table 2 only reports the parameter estimates for the first factor.^21^ First of all, note that testing for the joint in-sample significance of the factors, we reject the null regardless of *r* and *p*. Therefore, the factors have predictive power for IP. Comparing the ${\bar{R}}^2$’s obtained using the factors extracted by PC for $r=3$ and 7 with respect to those obtained for $r=1$, we can observe that adding more factors does not increase significantly the in-sample predictive performance of the regressions for IP.^22^ This result is in concordance with the conclusions in McCracken and Ng (2016), who interpret the first common factor (extracted using PC) as a real activity/employment factor. They find that the predictive information of the factors over IP changes over time with only the first common factor retaining its predictive information at the end of the sample period they consider. When $r=1$, the estimated parameters of the factor-augmented predictive regressions are very similar regardless of whether $p=1$ or 3 and the particular procedure used to extract the factors. Increasing the autoregressive lag of the factors and/or the number of factors only implies small improvements in the adjusted coefficient of determination with the best results obtained when the factors are extracted using ML-EM with $p=3$ if $r=1$ or 3. However, when $r=7$, the results are slightly better when the factors are extracted using KFS with the parameters of the DFM estimated using TS-LS.

**Table 4:** Adjusted coefficients of determination of factor-augmented predictive regressions based on factors estimated using PC, TS-LS and ML-EM

	$r=0$	$r=1$					$r=3$					$r=7$				
		PC	$p=1$		$p=3$		PC	$p=1$		$p=3$		PC	$p=1$		$p=3$	
			TS-LS	ML-EM	TS-LS	ML-EM		TS-LS	ML-EM	TS-LS	ML-EM		TS-LS	ML-EM	TS-LS	ML-EM
			*Jan. 1983–Dec. 2016*													
IP	0.20	0.27	0.27	0.27	0.27	0.30	0.28	0.28	0.29	0.28	0.30	0.33	0.34	0.34	0.37	0.35
Inflation	0.10	0.14	0.14	0.14	0.14	0.14	0.16	0.15	0.14	0.16	0.14	0.21	0.20	0.18	0.20	0.18
Employment	0.59	0.64	0.65	0.66	0.65	0.66	0.65	0.65	0.65	0.65	0.66	0.66	0.67	0.67	0.66	0.67
Income	0.00	0.12	0.13	0.15	0.13	0.15	0.14	0.15	0.16	0.15	0.17	0.15	0.15	0.16	0.16	0.16
			*Jan. 1983–Dec. 2007*													
IP	0.10	0.15	0.16	0.13	0.15	0.14	0.17	0.18	0.17	0.21	0.20	0.22	0.25	0.23	0.28	0.24
Inflation	0.15	0.15	0.15	0.15	0.15	0.15	0.15	0.15	0.15	0.15	0.15	0.19	0.20	0.20	0.19	0.20
Employment	0.48	0.53	0.53	0.51	0.53	0.52	0.52	0.52	0.51	0.52	0.51	0.53	0.55	0.54	0.55	0.54
Income	0.07	0.16	0.17	0.15	0.17	0.15	0.16	0.17	0.15	0.17	0.15	0.16	0.18	0.19	0.18	0.19

Table 4, which reports the ${\bar{R}}^2$ of the augmented predictive regressions corresponding to inflation, employment and income, shows that the conclusions are the same for these variables than those of IP. Note that McCracken and Ng (2016) interpret the third common factor as an inflation factor while the second common factor was dominated by forward-looking variables such as term interest rate spreads and inventories. They show that, in the sample period they consider, the first common factor does not have any predictive content for forecasting inflation in later times. Therefore, it seems that including the relevant number of factors could be relevant in in-sample prediction while the specification of the autoregressive lag could be of less importance. Furthermore, factor extraction based on KFS is slightly better than that based on PC and, unless the number of parameters is too large, it is better to estimate the parameters using EM.

### Out-of-sample forecasts

**Table 5:** One-step-ahead out-of-sample forecasts of first differences of industrial production, inflation, employment and income from January 2017 to December 2020, based on factor-augmented predictive regressions with factors estimated by PC, TS-LS and ML-EM

		$p=1$				$p=3$			
		MSFE (2019)	MSFE (2020)	Cov. 70% (2019)	Cov. 70% (2020)	MSFE (2019)	MSFE (2020)	Cov. 70% (2019)	Cov. 70% (2020)
		*Industrial production*							
$r=0$		0.55	4.23	77.78%	68.75%				
$r=1$	PC	0.61	5.62	72.22%	60.42%				
	TS-LS	0.51	3.16	80.56%	64.58%	0.51	3.24	80.56%	64.58%
	ML-EM	0.50	2.37	77.78%	64.58%	0.51	2.51	77.78%	66.67%
$r=3$	PC	0.60	5.09	75%	62.5%				
	TS-LS	0.51	3.17	77.78%	64.58%	0.52	3.25	72.22%	60.42%
	ML-EM	0.83	3.08	72.22%	62.5%	0.55	3.16	75%	62.5%
$r=7$	PC	0.53	4.57	75%	66.67%				
	TS-LS	0.51	4.55	77.78%	62.5%	0.55	4.77	77.78%	64.58%
	ML-EM	0.62	5.78	80.56%	64.58%	0.77	5.10	66.67%	54.17%
		*Inflation*							
$r=0$		0.35	0.90	94.44%	85.42%				
$r=1$	PC	0.34	0.91	94.44%	85.42%				
	TS-LS	0.36	0.84	91.67%	83.33%	0.35	0.84	91.67%	83.33%
	ML-EM	0.34	0.78	94.44%	85.42%	0.34	0.77	94.44%	85.42%
$r=3$	PC	0.33	0.89	94.44%	85.42%				
	TS-LS	0.35	0.90	94.44%	85.42	0.36	0.89	94.44%	85.42%
	ML-EM	0.38	1.03	94.44%	83.33%	0.38	0.99	94.44%	81.25%
$r=7$	PC	0.33	0.99	91.67%	83.33%				
	ML-EM	0.39	1.24	83.33%	77.08%	0.49	2.33	83.33%	75%
		*Employment*							
$r=0$		0.07	2.76	97.22%	79.17%				
$r=1$	PC	0.06	2.76	97.22%	81.25%				
	TS-LS	0.08	1.80	100%	85.42%	0.09	1.79	100%	85.41%
	ML-EM	0.09	1.51	100%	81.25%	0.09	1.45	100%	81.25%
$r=3$	PC	0.06	2.89	100%	81.25%				
	TS-LS	0.09	1.95	97.22%	81.25%	0.09	1.93	97.22%	81.25%
	ML-EM	0.22	1.91	86.11%	75%	0.18	1.87	91.67%	75%
$r=7$	PC	0.06	2.96	100%	83.33%				
	TS-LS	0.11	1.73	100%	87.5%	0.11	1.69	97.22%	83.33%
	ML-EM	0.27	2.94	88.89%	75%	0.91	8.68	52.78%	45.83%
		*Income*							
$r=0$		0.18	1.05	97.22%	85.42%				
$r=1$	PC	0.18	1.05	97.22%	79.17%				
	TS-LS	0.18	1.09	97.22%	87.5%	0.18	1.10	97.22%	87.5%
	ML-EM	0.19	1.04	97.22%	83.33%	0.19	1.09	97.22%	83.33%
$r=3$	PC	0.18	1.08	97.22%	79.17%				
	TS-LS	0.19	1.27	100%	89.58%	0.19	1.29	100%	89.58%
	ML-EM	0.22	1.09	97.22%	87.5%	0.21	1.11	97.22%	87.5%
$r=7$	PC	0.19	1.10	97.22%	77.08%				
	TS-LS	0.27	1.38	91.67%	83.33%	0.27	1.44	91.67%	81.25%
	ML-EM	0.37	1.96	88.89%	72.92%	0.99	5.71	61.11%	54.17%

The table reports the MSFE and the coverage of 70% forecast intervals computed with and without incorporating the forecasts corresponding to the year 2020

Finally, using the estimated factor-augmented regressions reported in Table 3 and the filtered factors obtained in the out-of-sample period, we obtain one-step-ahead forecasts of IP, inflation, employment and income from January 2017 to December 2020 and their corresponding 70% and 95% forecast intervals. We consider a fixed scheme with the parameters used for forecasting not being updated. Table 5 reports the empirical mean square forecast errors (MSFEs) and the empirical coverages of the 70% forecast intervals computed both with the forecasts obtained until December 2019 and until December 2020.^23^ Note that in the latter case, we are incorporating in the analysis the forecasts obtained during the turbulent times due to the recession induced by the COVID-19 pandemic, while, in the former case, the forecasts are obtained in a “normal” time in the evolution of the variables. The ratio between the out-of-sample and in-sample number of observations is $\frac{48}{396}=0.12$.

**Fig. 4:**
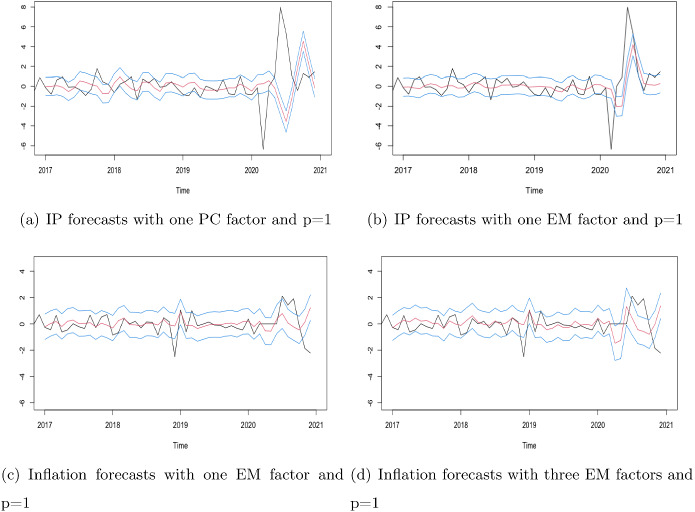
Out-of-sample forecasts of IP (first row) and inflation (second row) together with the corresponding 70% confidence intervals

**Table 6:** Tests of predictive ability of the factors when $r=1$

		IP		Inflation		Employment		Income	
		ENC	MSE	ENC	MSE	ENC	MSE	ENC	MSE
		Jan. 2017–Dec. 2019							
		Critical values ENC test: $90\%=0.776$, $95\%=1.06$							
		Critical values MSE test: $90\%=1.060$, $95\%=1.566$							
	PC	1.14**	$-3.40$	0.88*	1.07*	4.66**	6.31**	1.02*	$-0.44$
$p=1$	TS-LS	2.50**	3.04**	0.12	$-1.00$	1.64**	$-6.26$	2.11**	0.10
	ML-EM	3.32**	3.70**	0.71	0.59	6.22**	$-7.36$	1.52**	$-1.76$
$p=3$	TS-LS	2.36**	2.83**	0.13	$-1.00$	1.63**	$-6.29$	2.13**	0.05
	ML-EM	3.23**	3.43**	0.75	0.68	5.96**	$-6.17$	1.61**	$-1.43$
		Jan. 2017–Dec. 2020							
		Critical values ENC test: $90\%=0.776$, $95\%=1.06$							
		Critical values MSE test: $90\%=1.060$, $95\%=1.566$							
	PC	$-2.99$	$-11.86$	0.09	$-0.79$	2.11**	0.09	1.45**	0.27
$p=1$	TS-LS	12.06**	16.25**	2.90**	3.12**	17.61**	25.69**	4.31**	$-1.47$
	ML-EM	29.43**	37.52**	5.10**	6.99**	37.35**	39.70**	9.15**	0.25
$p=3$	TS-LS	11.12**	14.68**	2.89**	3.09**	17.65**	25.86**	4.21**	$-1.85$
	ML-EM	27.39**	33.04**	5.86**	8.02**	40.37**	43.11**	8.42**	$-1.75$
		Jan. 2008–Dec. 2019							
		Critical values ENC test: $90\%=1.528$, $95\%=2.181$							
		Critical values MSE test: $90\%=1.060$, $95\%=1.566$							
	PC	0.88	$-8.59$	$-1.36$	0.12	0.47	$-13.29$	$-0.13$	$-10.00$
$p=1$	TS-LS	$-2.29$	9.81**	$-0.25$	7.54**	3.68**	11.32**	$-0.12$	20.25**
	ML-EM	$-3.51$	13.47**	$-0.82$	6.66**	6.44**	13.13**	$-0.38$	16.47**
$p=3$	TS-LS	$-1.60$	9.80**	$-0.28$	7.59**	2.25**	10.99**	$-0.31$	20.10**
	ML-EM	$-3.23$	10.92**	$-0.85$	6.27**	5.65**	11.81**	$-0.46$	14.57**
		Jan. 2008–Dec. 2020							
		Critical values ENC test: $90\%=1.528$, $95\%=2.181$							
		Critical values MSE test: $90\%=1.060$, $95\%=1.566$							
	PC	$-1.83$	$-10.75$	0.07	0.06	$-4.08$	$-16.90$	$-3.69$	$-11.53$
$p=1$	TS-LS	8.44**	12.76**	5.68**	8.85**	10.43**	14.63**	15.20**	21.30**
	ML-EM	12.26**	16.07**	4.70**	7.58**	12.14**	16.85**	13.32**	17.6**
$p=3$	ST-LS	8.29**	12.64**	5.75**	8.90**	10.20**	14.28**	15.12**	21.15**
	ML-EM	10.85**	12.65**	4.38**	7.11**	10.89**	15.24**	11.83**	15.73**

*, ** Significant at 10% and 5 % levels, respectively

First of all, Table 5 shows that, even if the differences between the in-sample estimated factors and corresponding predictive regressions are minor, the performance of out-of-sample one-step-forecasts can be quite different. The procedure used to extract the factors and the estimator of the DFM parameters when the factors are extracted using the Kalman filter and smoother are relevant for the out-of-sample one-step-ahead forecasts performance. Table 6 reports the value of the ENC-F and MSE-F statistics to test the out-of-sample predictive ability of each of the factor-augmented predictive regressions considered when $r=1$ with respect to the AR(4) model without factors.^24^ Note that the asymptotic null distributions of ENC-F and MSE-F are not strictly positive and, consequently, the fact that the test values are sometimes negative does not necessarily constitute evidence in favor of the restricted model; see the explanations by Stauskas and Westerlund (in press). Table 6 shows that the ENC-F tests support that the factors are significative to forecast IP, employment and income. However, out-of-sample forecasts of inflation are only significantly different from the forecasts obtained with the AR(4) model without factors at the 90% level. In general, the ENC-F statistic is largest when the factor is extracted using KFS with the parameters estimated by ML-EM. However, the MSE-F test is more conservative and, except for IP, it generally rejects that the out-of-sample MSFEs are reduced by introducing a factor. Nevertheless, the differences between procedures used to extract the factors are more obvious when there are extraordinary movements in the series, as those observed during the COVID-19 crisis; see Fig. 4. When we take into account 2020, the differences in the MSFE obtained with respect to PC are striking (for instance, for IP, the out-of-sample MSFE based on the PC extracted factors is more than twice that of the forecasts based on ML extracted factors). However, removing the year 2020 gives very different numerical results. First, the magnitude of the MSFEs is considerably reduced. In particular, PC induced MSE is around 10 times smaller when excluding 2020. Nevertheless, the PC extracted factors still renders out of sample MSFEs around 20% larger than those of KFS methods. Regarding the length of the AR polynomial of the common factor for IP, notice that we always obtain smaller MSEs for $p=1$, that is, the shorter the memory of the common factor, the smaller the MSFE. According to our results, forecasts of inflation based on models with $r=1$ or $r=3$ are different; see Fig. 4. On top of the noticeable differences between results including pre-COVID times and those that do not include them that we can also observe with three factor models, notice that both for IP and inflation including more factors does not necessarily translate into smaller out- of-sample MSFEs; see also Barhoumi et al. (2013), who also conclude that increasing the number of factors do not decrease MSFEs when forecasting French and German GDP. Indeed, in occasions those are larger than the corresponding ones from one factor predictive regressions. In general, regardless of the variable to be forecast, the out-of-sample performance is better for those forecasts based on models with smaller number of factors and smaller autoregression lags extracted using KFS and with the parameters estimated using TS-LS. Our results seem to support the KISS (Keep it sophistically simple) principle.

**Table 7:** Parameter estimates of factor-augmented predictive regressions for IP based on factors estimated using PC, TS-LS and ML-EM

	$r=0$	$r=1$					$r=3$					$r=7$				
		PC	$p=1$		$p=3$		PC	$p=1$		$p=3$		PC	$p=1$		$p=3$	
			TS-LS	ML-EM	TS-LS	ML-EM		TS-LS	ML-EM	TS-LS	ML-EM		TS-LS	ML-EM	TS-LS	ML-EM
$\mu$	$-0.013$	$-0.012$	$-0.011$	$-0.013$	$-0.010$	$-0.013$	$-0.010$	$-0.007$	.0.007	$-0.005$	$-0.006$	$-0.011$	$-0.009$	$-0.009$	$-0.008$	$-0.008$
	(0.81)	(0.83)	(0.84)	(0.81)	(0.85)	(0.80)	(0.85)	(0.90)	(0.89)	(0.92)	(0.91)	(0.83)	(0.86)	(0.87)	(0.86)	(0.88)
$\delta _1$	$-0.000$	$-0.280$	$-0.373$	$-0.102$	$-0.326$	$-0.063$	$-0.192$	$-0.288$	0.027	$-0.172$	0085	0.265	0.536	0.364	0.531	0.389
	(1.00)	(0.06)	(0.00)	(0.61)	(0.00)	(0.75)	(0.14)	(0.06)	(0.90)	(0.25)	(0.68)	(0.15)	(0.01)	(0.03)	(0.01)	(0.02)
$\delta _2$	0.206	$-0.021$	$-0.084$	$-0.421$	$-0.113$	$-0.466$	$-0.139$	$-0.271$	$-0.519$	$-0.308$	$-0.565$	$-0.159$	$-0.322$	$-0.151$	$-0.372$	$-0.184$
	(0.00)	(0.84)	(0.46)	(0.03)	(0.32)	(0.02)	(0.28)	(0.07)	(0.01)	(0.03)	(0.01)	(0.39)	(0.15)	(0.36)	(0.09)	(0.26)
$\delta _3$	0.198	0.213	0.206	$-0.124$	0.189	$-0.190$	0.022	0.001	$-0.249$	$-0.066$	$-0.333$	$-0.011$	$-0.103$	0.059	$-0.159$	0.046
	(0.00)	(0.03)	(0.07)	(0.53)	(0.09)	(0.33)	(0.86)	(0.99)	(0.24)	(0.65)	(0.10)	(0.95)	(0.64)	(0.71)	(0.46)	(0.77)
$\delta _4$	0.089	0.229	0.269	0.333	0.271	0.331	0.266	0.362	0.298	0.372	0.305	0.360	0.461	0.383	$-0.452$	0.399
	(0.13)	(0.02)	(0.01)	(0.09)	(0.01)	(0.09)	(0.04)	(0.02)	(0.17)	(0.01)	(0.14)	(0.05)	(0.03)	(0.02)	(0.03)	(0.01)
$\beta _{11}$	–	0.387	0.509	0.097	0.436	0.050	0.400	0.506	0.091	0.445	0.068	0.007	$-0.203$	$-0.262$	$-0.239$	$-0.381$
	–	(0.00)	(0.00)	(0.66)	(0.00)	(0.82)	(0.00)	(0.00)	(0.69)	(0.01)	(0.76)	(0.97)	(0.33)	(0.22)	(0.24)	(0.08)
$\beta _{12}$	–	0.288	0.350	0.704	0.411	0.759	0.350	0.556	0.915	0.607	0.985	0.228	0.421	0.43	0.539	0.606
	–	(0.04)	(0.03)	(0.00)	(0.01)	(0.00)	(0.01)	(0.00)	(0.00)	(0.00)	(0.00)	(0.20)	(0.07)	(0.09)	(0.02)	(0.02)
$\beta _{13}$	–	$-0.118$	$-0.129$	0.319	$-0.106$	0.390	$-0.101$	$-0.198$	0.153	$-0.225$	0.147	$-0.081$	$-0.060$	$-0.021$	$-0.068$	$-0.075$
	–	(0.39)	(0.41)	(0.13)	(0.49)	(0.06)	(0.47)	(0.27)	(0.52)	(0.19)	(0.52)	(0.65)	(0.79)	(0.93)	(0.76)	(0.77)
$\beta _{14}$	–	$-0.296$	$-0.379$	$-0.320$	$-0.389$	$-0.326$	$-0.246$	$-0.338$	$-0.254$	$-0.308$	$-0.218$	$-0.111$	$-0.220$	$-0.423$	$-0.168$	$-0.440$
	–	(0.02)	(0.01)	(0.137)	(0.01)	(0.13)	(0.07)	(0.04)	(0.26)	(0.06)	(0.32)	(0.53)	(0.29)	(0.04)	(0.42)	(0.04)
$\sigma _u$	0.947	0.922	0.915	0.928	0.918	0.923	0.911	0.901	0.910	0.886	0.892	0.88	0.862	0.874	0.847	0.868
${\bar{R}}^2$	0.10	0.15	0.16	0.13	0.15	0.14	0.17	0.18	0.17	0.21	0.20	0.22	0.25	0.23	0.28	0.24

*p*-values in parenthesis. Data up to December 2007

Table 5, which also reports the empirical coverages of the 70% forecast intervals, shows that these intervals are usually too large with coverages well above the nominal. The reason for this empirical observation deserves further investigation, which is beyond our objectives in this paper.

### Robustness check: forecasting over different periods of time

It is well known that, when forecasting in practice, the use of different window sizes for the out-of-sample forecasts may lead to different empirical results. It is possible that, for a given forecast window, significant predictive ability is not detected while it could be detected in another window. On the other hand, it is also possible to obtain satisfactory results just by chance. Moreover, the results on the ability of predictive models relies on the ratio between the out-of-sample and in-sample observations with the predictive tests, ENC-F and MSE-F, being more accurate when *H* is large. Consequently, in this subsection, we study the robustness of the empirical results above to the choice of estimation and out-of-sample window sizes. In particular, the parameters are estimated using data up to December 2007, so that the in-sample period does not include data from the last global financial crisis. In this case, the estimation size is $T=288$ while the out-of-sample forecast size is $H=156$. Therefore, $\frac{\sqrt{T}}{N}=0.13$ and a ratio of out-of-sample to in-sample observations of 0.54.

Table 7 reports the parameter estimates of the factor-augmented predictive regressions for IP obtained using the in-sample data from January 1983 to December 2007 while Table 4 reports the ${\bar{R}}^2$ of the regressions not only of IP but also of inflation, employment and income.^25^ Looking at the results for IP in Table 7, we can observe that the conclusions are mostly the same as those obtained when the regressions were estimated with data up to December 2016. The factors are significant and, although the fit, measured by the adjusted coefficient of determination, is smaller than those reported in Table 3, it is maximized when the predictive regressions are estimated including 7 factors estimated using KFS and specified as a VAR(3).

With respect to the in-sample fit of the predictive regressions of inflation estimated with data up to December 2007, Table 4 shows that it is very similar to that reported for the regressions estimated with data up to December 2016. The only difference observed is that in this latter case, the factors are not even significant to forecast inflation. The results for employment are very similar to those described for IP. Finally, when looking at the ${\bar{R}}^2$ coefficients of the predictive regressions for real income, we observe that they are slightly larger than those of the models estimated using data up to December 2016 but still support the main conclusion about being significant when the factors are included to forecast and maximized if 7 factors extracted using KFS and modeled as VAR(3) are considered. All in all, the main conclusions from the in-sample analysis are supported using this alternative estimation window.

Finally, Table 8 reports the MSFEs and coverages of the out-of-sample forecasts obtained from January 2008 until either December 2019 or December 2020. We can observe that the factors have predictive power if they are extracted using KFS; see also the results of the predictive ability tests in Table 6. As above, when considering the forecasts since January 2017, the results are stronger when the COVID19 pandemic year, 2020, is included in the out-of-sample period.

**Table 8:** One-step-ahead out-of-sample forecasts of first differences of industrial production, inflation, employment and income based on factor-augmented predictive regressions with factors estimated by: PC, TS-LS and ML-EM

		$p=1$				$p=3$			
		MSFE (2019)	MSFE (2020)	Cov. 70% (2019)	Cov. 70% (2020)	MSFE (2019)	MSFE (2020)	Cov. 70% (2019)	Cov. 70% (2020)
		*Industrial production*							
$r=0$		0.85	1.90	79.86%	77.56%				
$r=1$	PC	1.03	2.40	77.78%	73.72%				
	TS-LS	0.73	1.49	80.56%	77.56%	0.74	1.52	81.94%	78.85%
	ML-EM	0.72	1.75	82.64%	78.84%	0.75	1.79	81.25%	77.56%
$r=3$	PC	1.13	2.30	77.08%	75.64%				
	TS-LS	0.74	1.56	77.08%	73.72%	0.74	1.64	77.78%	74.36%
	ML-EM	0.72	1.99	79.86%	76.92%	0.75	2.00	78.47%	75.64%
$r=7$	PC	0.98	1.82	75.69%	73.72%				
	TS-LS	0.79	2.12	77.78%	73.72%	0.81	2.18	76.39%	71.79%
	ML-EM	0.72	2.04	79.17%	76.92%	0.93	2.35	78.47%	75%
		*Inflation*							
$r=0$		0.53	0.71	84.72%	82.69%				
$r=1$	PC	0.53	0.71	84.72%	82.69%				
	TS-LS	0.49	0.70	86.11%	83.97%	0.49	0.70	86.11%	83.97%
	ML-EM	0.49	0.67	84.03%	82.05%	0.50	0.67	84.03%	82.05%
$r=3$	PC	0.54	0.73	84.03%	82.05%				
	TS-LS	0.52	0.75	86.81%	84.62%	0.52	0.76	86.81%	84.62%
	ML-EM	0.51	0.72	86.11%	83.97%	0.51	0.72	86.11%	83.97%
$r=7$	PC	0.60	0.82	81.94%	79.49%				
	TS-LS	0.48	0.72	85.42%	83.33%	0.48	0.71	84.72%	82.69%
	ML-EM	0.56	0.78	85.42%	82.69%	0.61	0.88	82.29%	82.41%
		*Employment*							
$r=0$		0.28	1.20	90.97%	86.53%				
$r=1$	PC	0.4	1.35	87.5%	82.69%				
	TS-LS	0.23	0.87	91.67%	87.82%	0.23	0.87	91.67%	87.82%
	ML-EM	0.23	0.90	98.61%	94.87%	0.23	0.91	91.67%	87.18%
$r=3$	PC	0.41	1.38	86.81%	82.05%				
	TS-LS	0.23	0.92	92.36%	92.95%	0.23	0.91	92.36%	88.46%
	ML-EM	0.23	0.98	90.97%	86.54%	0.23	0.96	92.36%	87.82%
$r=7$	PC	0.84	1,82	81.25%	76.92%				
	TS-LS	0.21	0.71	94.44%	90.38%	0.21	0.69	93.75%	90.38%
	ML-EM	0.51	1.05	83.33%	80.13%	0.91	1.37	75%	71.79%
		*Income*							
$r=0$		1.07	1.32	95.14%	92.95%				
$r=1$	PC	1.33	1.58	78.47%	73.08%				
	TS-LS	0.94	1.17	80.56%	77.56%	0.94	1.17	80.56%	77.56%
	ML-EM	0.95	1.20	68.75%	72.22%	0.97	1.21	77.78%	75.64%
$r=3$	PC	1.39	1.64	77.78%	72.44%				
	TS-LS	0.95	1.21	79.86%	76.92%	0.95	1.21	79.86%	76.92%
	ML-EM	0.99	1.27	79.86%	76.92%	1.03	1.29	78.47%	75.64%
$r=7$	PC	1.62	1.88	72.22%	67.95%				
	TS-LS	1.02	1.26	77.08%	75.64%	1.02	1.27	78.47%	76.92%
	ML-EM	1.04	1.29	73.61%	71.15%	1.21	1.56	75%	71.15%

The table reports the MSFE, with *p*-values for equal predictive ability with respect to the benchmark AR(4) in parenthesis, and the coverage of 70% forecast intervals. Both quantities have been computed with and without incorporating the predictions corresponding to the year 2020. Forecasts from January 2008

## Conclusions

The factors are highly correlated among them regardless of the procedure or estimator used for their extraction and the number of lags specified for their autoregressions. However, the main differences between factor estimates obtained using PC or KFS based on either TS-LS or ML-EM are observed in their dynamics and these differences may have implication in forecasting. In the particular US macroeconomic data set analyzed in this paper, the largest autoregressive root is closer to one when the factor is extracted using the KFS algorithm with the DFM’s parameters estimated by ML-EM. The likelihood-ratio tests of the DFMs favor specifications with more factors and more lags. Furthermore, the same conclusion is obtained when looking at the results of the in-sample factor-augmented predictive regressions, which have larger fit measures when the factors are extracted using KFS from DFMs with large number of factors modeled as VAR(*p*) processes with $p>1$. With respect to the estimator of the parameters of the DFM, the results are better if the ML-EM estimator is used when the number of parameters to be estimated is not very large. However, if the number of parameters is large, the ML-EM estimator seems to have numerical problems and, consequently, the fit of the factor-augmented predictive regressions is better when the parameters are estimated using the simpler TS-LS estimator. Finally, according to our empirical results, we show that increasing the number of factors and/or their lag structure does not always lead to an increase in the out-of-sample forecast precision. The out-of-sample MSFEs are generally minimized when forecasts are based on simple models with one factor extracted using KFS and modelled as an AR(1) process. This conclusion is rather general for the four variables considered for forecasting in this paper. In any case, answering the question in the title of this paper, a careful specification of the DFM before factor extraction could be important in terms of in-sampling fitting. However, when forecasting out-of-sample simple specifications seem to be favored.

## Footnotes

1 In this paper, we focus on models representing comovements in the first order moments of the variables in the system of interest. However, there are also proposals in the literature to model common movements in other characteristics of the distribution; see Chen et al. (2021) for a factor model with the factors being common in the quantiles.

2 We assume that all deterministic components have been removed from the series $y_{it}$ previous to their analysis.

3 Bai and Ng (2013) derive the limiting distribution of PC factors under alternative identification restrictions.

4 The optimization algorithm used in the empirical application of this paper is a quasi-Newton–Raphson algorithm as implemented in the subroutine *Optim* in R.

5 These triangular restrictions link the first factor with the first variable in the system, the second with the first two variables and so on. However, note that, as mentioned above, the estimated matrix of loadings can always be rotated to give alternative interpretations of the factors.

6 Note that, if the VAR order is $p>1$, the EM estimator can be easily modified.

7 The estimates $f^{(0)}_{t|T}$ of this first iteration are usually known as two-step LS (TS-LS) estimates of the factors; see Doz et al. (2011). In this case, the covariance matrix of $u_t$ is estimated by

$$\begin{aligned} {\hat{\Sigma }}_u^{(0)}=\frac{1}{T}\sum _{t=1}^T{\tilde{u}}_t{\tilde{u}}_t^{\prime } \end{aligned}$$

where $${\tilde{u}}_t={\tilde{f}}_{t}^{\mathrm{PC}}-{\hat{\Phi }}^{(0)} {\tilde{f}}_{t-1}^{\mathrm{PC}}$$.

8 Note that Bai and Li (2016) propose using $\Lambda ^{(1)}$ instead of $\Lambda ^{(0)}$ to calculate the idiosyncratic residuals.

9 The resulting estimator is known in the related literature as Quasi-ML (QML).

10 In the context of inference for the LS estimator of the parameters of factor-augmented predictive regression models, Gonçalves and Perron (2014) show that the finite sample properties of the asymptotic distribution can be poor, especially if the cross-sectional dimension is not sufficiently large relative to the temporal dimension.

11 The asymptotic variance is obtained using the usual formulae for regression models, adding an additional additive term due to the estimation of the factors. However, if *N* is large, this additional term disappears.

12 These critical values are valid under the assumption of homoscedasticity of the forecast errors. Clark and McCracken (2012) and Hansen and Timmermann (2015) derive critical values under conditional heteroscedasticity. Very recently, Stauskas and Westerlund (in press) propose an extension of the tests proposed by Gonçalves et al. (2017) that takes into account that the number of factors is unknown. We were not aware of this extension at the moment of writing the paper.

13 This data base is an updated version of the data base used by Stock and Watson (2002a) and Stock and Watson 2012. Although data are available from January 1959, there is a generalized consensus about the presence of a structural break in 1982 due to the change of policy rule of the Federal Reserve which swichted from targeting non-borrowed reserves to targeting the federal funds rate; see, for example, Hallin and Liska (2007) and Luciani (2015). To avoid the problems associated to the presence of structural breaks when determining the number of factors, we analyze the data set observed from 1983; see Breitung and Tenhofen (2011b) who show that, in the presence of a structural break, the number of factors is overestimated and the factor loadings are inconsistently estimated, and Chen et al. (2014) for a test for breaks in DFMs.

14 Note that PC factor extraction is nonparametric and, consequently, it does not rely on any particular value of *p*.

15 Note that for the results to be comparable, all estimated loadings and factors have been rotated to the same base as those estimated by PC; see Poncela and Ruiz (2015) for the rotation. This rotation is needed because of the different restrictions used for identification by PC and KFS. While under PC the sample variance of the factors is assumed to be one, the restriction imposed when estimating by ML based on the KF is $\sigma ^2_u=1$. The ML estimate of the autoregressive parameter, ${\hat{\phi }}_1=0.87$ implies that the factor has variance 4.11. Therefore, the ML weights should be divided by 2.03 for the variance of the common component to be the same.

16 The confidence intervals of the PC factors are based on the asymptotic distribution derived by Bai (2003).

17 The intervals for the factors could be more realistic if the asymptotic MSE is modified by subsampling as proposed by Maldonado and Ruiz (forthcoming).

18 In this case, it is infeasible to maximize numerically the likelihood. Consequently, the ML estimates of the parameters are only obtained using the EM algorithm.

19 Note, however, that the MSE still doubles that obtained for the PC factors, which is 0.01.

20 The off-diagonal elements of the estimated autoregressive matrices are fixed to zero as, in practice, they are not far from zero.

21 The results for the other variables as well as residual diagnosis analysis of the factor augmented predictive regressions are available upon request.

22 A word of caution is due. In this paper, we include in each of the factor-augmented predictive regression all factors considered together with their corresponding four lags in a systematic way. However, one can also select the combinations of factors and/or lags with higher predictive content as in, for example, Gonçalves et al. (2017) and Demetrescu and Hacioglu Hoke (2019). Alternatively, one could extract the relevant factors taking into account that the objective is to use them as predictors for the variable $y_t$ and using, for example, Partial Least Squares (PLS); see the discussion by Ng (2013) and the application by Fuentes et al. (2015).

23 Note that these quantities are merely descriptive as they are based on just 36 forecasts in the former case and 48 in the latter. Results for intervals with 95% nominal coverage are also available upon request.

24 We do not report the statistics for $r=3$ and 7 as, in these cases, the critical values are not available. Furthermore, note that the critical values reported in Table 6 have been obtained using a rolling window scheme while we are using a fixed window. Therefore, they should be interpreted as approximations.

25 Note that the number of factors determined by each of the criteria considered are the same as those determined in the longer in-sample period analyzed before and reported in Table 1.
